# Shared PKS Module in Biosynthesis of Synergistic Laxaphycins

**DOI:** 10.3389/fmicb.2020.578878

**Published:** 2020-09-16

**Authors:** Lassi Matti Petteri Heinilä, David P. Fewer, Jouni Kalevi Jokela, Matti Wahlsten, Anna Jortikka, Kaarina Sivonen

**Affiliations:** Department of Microbiology, Faculty of Agriculture and Forestry, University of Helsinki, Helsinki, Finland

**Keywords:** synergy, biosynthesis, natural product, laxaphycin, scytocyclamide, antifungal, cupin, dehydrobutyrine

## Abstract

Cyanobacteria produce a wide range of lipopeptides that exhibit potent membrane-disrupting activities. Laxaphycins consist of two families of structurally distinct macrocyclic lipopeptides that act in a synergistic manner to produce antifungal and antiproliferative activities. Laxaphycins are produced by range of cyanobacteria but their biosynthetic origins remain unclear. Here, we identified the biosynthetic pathways responsible for the biosynthesis of the laxaphycins produced by *Scytonema hofmannii* PCC 7110. We show that these laxaphycins, called scytocyclamides, are produced by this cyanobacterium and are encoded in a single biosynthetic gene cluster with shared polyketide synthase enzymes initiating two distinct non-ribosomal peptide synthetase pathways. The unusual mechanism of shared enzymes synthesizing two distinct types of products may aid future research in identifying and expressing natural product biosynthetic pathways and in expanding the known biosynthetic logic of this important family of natural products.

## Introduction

Natural products are small molecules produced by living organisms (Newman and Cragg, 2016). Research interest in natural products is focused on the discovery of new molecules with pharmaceutical applications (Spainhour, 2005; Newman and Cragg, 2016). Natural products often have complex chemical structures with rare chemical moieties that allow them to react with specific molecular targets and to kill or inhibit the growth of other organisms (Rodrigues et al., 2016). Cyanobacteria produce a wide variety of natural products with potent bioactivities (Demay et al., 2019; Huang and Zimba, 2019). Characterization of new natural products offers starting material for drug design as new active structures (Rodrigues et al., 2016). Characterization of the biosynthesis of these products advance methods in production of the structures through combinatorial biosynthesis (Kim et al., 2015). Many microbial and cyanobacterial natural products are synthesized by polyketide synthases (PKS) and non-ribosomal peptide synthetases (NRPS) (Kehr et al., 2011; Dittmann et al., 2015). PKS and NRPS enzymes often act together and are encoded in joint biosynthetic gene clusters producing hybrid PKS/NRPS products (Miyanaga et al., 2018). PKS and NRPS enzymes allow the production of complex structures with characteristic non-proteinogenic amino acids and the combination of non-ribosomal peptides (NRP) with polyketide chains and decorations (Evans et al., 2011). NRPS and PKS biosynthesis typically follows a colinearity rule, where the number and order of the catalytic domains correspond to the amino acid number, order and structure in the product (Guenzi et al., 1998; Callahan et al., 2009).

Laxaphycins are cyanobacterial cyclic lipopeptides that fall in two distinct structural macrocycles consisting of either 11 amino acids (known as A-type laxaphycins) or 12 amino acids (known as B-type laxaphycins) (Frankmölle et al., 1992a; Luo et al., 2015). Both types include β-aminooctanoic acid (Aoa) or β-aminodecanoic acid (Ada) (Table 1). Eleven- and 12-residue laxaphycins have strong synergistic activity in antifungal and antiproliferative bioactivity assays (Frankmölle et al., 1992b; MacMillan et al., 2002; Cai et al., 2018). Laxaphycins are hypothesized to be produced by the PKS/NRPS hybrid pathway (Bornancin et al., 2015, 2019). However, the biosynthetic origins of members of the laxaphycin family remains unclear. Despite sharing the same name, they are chemically distinct and are anticipated to be produced by distinct pathways (Bornancin et al., 2015, 2019). The nomenclature of laxaphycins is complicated due to the two distinct core types addressed as a single family combined with naming new members after the producing organisms and distinguishing variants with lettering complicates (Table 1). Therefore, we refer to the two types as 11- and 12-residue laxaphycins. There are 30 diverse members assigned to the laxaphycin family reported to date (Table 1). The first laxaphycins to exhibit synergistic effects were described from *Anabaena laxa* (Frankmölle et al., 1992a,b). Here, we focused on laxaphycin variants called scytocyclamides produced by *Scytonema hofmannii* PCC 7110 (Grewe, 2005). *S. hofmannii* PCC 7110 was previously studied by our group and a methanol crude extract of the cells was antifungal but the active agent was not identified (Shishido et al., 2015).

**TABLE 1:** Amino acid sequence of laxaphycin variants.

	**Amino acid residue**												**Ref.**

**11-residue laxaphycins**	**1**	**2**	**3**	**4**	**5**	**6**	**7**	**8**	**9**	**10**	**11**		
Laxaphycin A	Aoa	Hse	Dhb	OHPro	HSe	Phe	Leu	Ile	Ile	Leu	Gly		1
Laxaphycin A2	Aoa	Hse	Dhb	OHPro	HSe	Phe	Leu	Val	Ile	Leu	Gly		10
Laxaphycin E	Ada	Hse	Dhb	OHPro	HSe	Phe	Leu	Ile	Ile	Leu	Gly		1
Hormothamnin A	Aoa	Hse	Dhb	OHPro	HSe	Phe	Leu	Ile	Ile	Leu	Gly		2
Lobocyclamide A	Aoa	Ser	Dhb	OHPro	HSe	Tyr	Leu	Ile	Ile	Leu	Gly		3
Trichormamide A	Ada	Ser	Ser	Pro	Ser	Tyr	Leu	Ile	Ile	Pro	Gly		7
Trichormamide D	Ada	Gln	Dhb	Pro	Ser	Tyr	Leu	Val	Phe	Leu	Gly		8
Scytocyclamide A	Aoa	Gln	Dhb	OHPro	HSe	Phe	Leu	Ile	Ile	Leu	Gly		4
[L-Val^8^]laxaphycin A	Aoa	Hse	Dhb	OHPro	HSe	Phe	Leu	Val	Ile	Leu	Gly		11
[D-Val^9^]laxaphycin A	Aoa	Hse	Dhb	OHPro	HSe	Phe	Leu	Ile	Val	Leu	Gly		11
Acyclolaxaphycin A	H-Aoa	Hse	Dhb	OHPro	HSe	Phe	Leu	Ile	Ile	Leu	Gly-OH		11
[des-Gly^11^] acyclolaxaphycin A	H-Aoa	Hse	Dhb	OHPro	HSe	Phe	Leu	Ile	Ile	Leu-OH			11
[des-(Leu^10^-Gly^11^)] acyclolaxaphycin A	H-Aoa	Hse	Dhb	OHPro	HSe	Phe	Leu	Ile	Ile-OH				11

**12-residue laxaphycins**	**1**	**2**	**3**	**4**	**5**	**6**	**7**	**8**	**9**	**10**	**11**	**12**	**Ref.**

Laxaphycin B	Ada	Val	OHLeu	Ala	OHLeu	Gln	NMe-Ile	OHAsn	Thr	Pro	Leu	Thr	1
Laxaphycin B2	Ada	Val	OHLeu	Ala	Leu	Gln	NMe-Ile	OHAsn	Thr	Pro	Leu	Thr	5
Laxaphycin B3	Ada	Val	OHLeu	Ala	OHLeu	Gln	NMe-Ile	OHAsn	Thr	OHPro	Leu	Thr	5
Laxaphycin B4	Ada	Val	OHLeu	Hse	OHLeu	Gln	NMe-Ile	OHAsn	Thr	OHPro	Leu	Thr	10
Laxaphycin B5	Ada	Ile	OHLeu	Val	OHLeu	Gln	NMe-Ile	Asn	Thr	Pro	Tyr	Thr	12
Laxaphycin B6	Ada	Ile	OHLeu	Val	Leu	Gln	NMe-Ile	Asn	Thr	Pro	Tyr	Thr	12
Laxaphycin D	Aoa	Val	OHLeu	Ala	OHLeu	Gln	NMe-Ile	OHAsn	Thr	Pro	Leu	Thr	1
Lobocyclamide B	Ada	Val	OHLeu	Ala	OHLeu	Gln	NMe-Ile	OHThr	Thr	OHPro	Leu	Thr	3
Lobocyclamide C	Aoa	Val	OHLeu	Ala	OHLeu	Gln	NMe-Ile	OHThr	Thr	OHPro	Leu	Thr	3
Lyngbyacyclamide A	Ada	Val	OHLeu	Hse	Leu	Gln	NMe-Ile	OHAsn	Thr	Pro	Phe	Thr	6
Lyngbyacyclamide B	Ada	Val	OHLeu	Hse	Leu	Gln	NMe-Ile	OHAsn	Thr	OHPro	Phe	Thr	6
Trichormamide B	Ada	Ile	OHLeu	Hse	OHLeu	Gln	NMe-Ile	Ser	Thr	Pro	Tyr	Thr	7
Trichormamide C	Ada	Val	OHLeu	Ala	OHLeu	Gln	NMe-Ile	Asn	Thr	Pro	Leu	Thr	8
Acyclolaxaphycin B	Ada	Val	OHLeu-OH	H-Ala	OHLeu	Gln	NMe-Ile	OHAsn	Thr	Pro	Leu	Thr	9
Acyclolaxaphycin B3	Ada	Val	OHLeu-OH	H-Ala	OHLeu	Gln	NMe-Ile	OHAsn	Thr	OHPro	Leu	Thr	9
Scytocyclamide B	Aoa	Val	OHLeu	Ala	OHLeu	Gln	NMe-Ile	OHAsn	Thr	Pro	Leu	Thr	4
Scytocyclamide C	Aoa	Val	OHLeu	Ala	Leu	Gln	NMe-Ile	OHAsn	Thr	Pro	Leu	Thr	4

*1 Frankmölle et al. (1992b), 2 Gerwick et al. (1992), 3 MacMillan et al. (2002), 4 Grewe (2005), 5 Bonnard et al. (2007), 6 Maru et al. (2010), 7 Luo et al. (2014), 8 Luo et al. (2015), 9 Bornancin et al. (2015), 10 Cai et al. (2018), 11 Bornancin et al. (2019), 12 Sullivan et al. (2020). Aoa – β-aminooctanoic acid, Ada - β-aminodecanoic acid, Hse - Homoserine, Dhb - Dehydrobutyrine, NMe-Ile – N-Methyl Isoleucine, OHPro – 4-hydroxyproline, OHAsn – 3-hydroxyasparagine, OHLeu – 3-hydroxyleucine, OHThr – 4-hydroxythreonine.*

Here we describe the biosynthetic pathways responsible for the biosynthesis of scytocyclamides from *S. hofmannii* PCC 7110. We show that the two types of scytocyclamides are synthesized by a branched NRPS/PKS biosynthetic pathway. These pathways encode shared loading PKS enzymes that initiate two distinct NRPS pathways exceptionally to the colinearity rule. We also report the synergistic antifungal activity of scytocyclamides and three new laxaphycin variants (scytocyclamides A2, B2, and B3).

## Materials and Methods

### Scytocyclamide Purification

*Scytonema hofmannii* PCC 7110 was grown in 5-L Erlenmeyer flasks with 2.7 L modified Z8 medium without source of combined nitrogen (Supplementary Table S1) at 20–21°C with photon irradiation of 3–7 μmol m^−2^ s^−1^ with constant sterilized air bubbling for 3–5 weeks. Cells were collected by decanting excess media and centrifugation at 8000 × g for 5 min. Cells were frozen at −80°C and freeze-dried with CHRIST BETA 2–8 LSC plus freeze drier with a LYO CUBE 4–8 chamber. The total amount of freeze-dried biomass was 4 g.

For each gram of dry cells, 30 ml of methanol was used and the mixture was homogenized with Heidolph Silent crusher M at 20 000 rpm for 30 s. The suspension was centrifuged 10,000 × g for 5 min and supernatant was collected. Extraction of the precipitate was repeated with 30 ml of methanol. Chromatorex (Fuji-Davison Chemical Ltd., Aichi, Japan) chromatography silica ODS powder (10 ml) was added to the supernatant pool and the mixture was dried with rotary evaporator Büchi Rotavapor R-200 at 30°C. Solid phase extraction (SPE) was performed with Phenomenex SPE strata SI-1 silica 5 g/20 ml column, preconditioned with 20 ml isopropanol and 20 ml of heptane. Silica ODS powder with the dry extract was added to the column and extracted with heptane, ethyl acetate, acetone, acetonitrile, and methanol with each fraction collected individually. Fractions were dried with nitrogen gas flow and re-dissolved in 1 ml of methanol for bioactivity assays. The active methanol fraction was further fractionated with liquid chromatography. Chromatography was performed with an Agilent 1100 Series liquid chromatograph with a Phenomenex Luna 5 μm C18(2) (150 × 10 mm, 100 Å) column. The sample was injected in 100-μl batches and eluted with acetonitrile/isopropanol 1:1 (solvent B) and 0.1% HCOOH (solvent A) with a flow rate of 3 ml min^–1^ in the following four stages: 1, isocratic stage of 43% solvent B in A for 15 min; 2, a linear gradient of solvent B from 43% to 60% in 10 min; 3, a linear gradient of solvent B from 60% to 81% in 5 min; and 4, a linear gradient of solvent B from 81% to 100% in 6 min. Six scytocyclamide fractions were collected, dried with nitrogen, and weighed.

### 3-Hydroxyleucine Feeding Experiment

*Scytonema hofmannii* PCC 7110 was grown in 100-mL Erlenmeyer flasks with 41 mL modified Z8 medium without a source of combined nitrogen with 40 μM of racemic 3-OHLeu mixture of all four isomers (2-Amino-3-hydroxy-4-methylpentanoic acid, ABCR) to determine if 3-OHLeu is utilized as a substrate in scytocyclamide production. Control cultivations were grown on the same medium without added 3-OHLeu. For both media, three duplicates were cultivated at 20–21°C with photon irradiation of 3–7 μmol m^−2^ s^−1^ for 17 days. Cells were collected by decanting excess media and centrifugation 8000 × g for 5 min. Cells were frozen at −80°C and freeze-dried with CHRIST BETA 2–8 LSC plus with a LYO CUBE 4–8 freeze drier. Freeze-dried biomass was weighed and extracted with 0.5 ml methanol and glass beads (0.5-mm glass beads, Scientific Industries Inc., United States) using a FastPrep cell disrupter two times for 25 s at a speed of 6.5 m s^−1^. Samples were centrifuged at room temperature for 5 min at 10 000 × g and supernatant was collected.

### Peptide Identification by LC-MS

*Scytonema hofmannii* PCC 7110 was grown in 500-mL Erlenmeyer flasks of with 250 mL modified Z8 medium without a source of combined nitrogen at 20–21°C with photon irradiation of 3–7 μmol m^−2^ s^−1^ with constant sterilized air bubbling for 4 weeks. Cells were collected by decanting excess media and centrifugation at 8000 × g for 5 min. Cells were frozen at −80°C and freeze-dried with CHRIST BETA 2–8 LSC plus with a LYO CUBE 4–8 freeze drier. Freeze-dried cells (100 mg) were extracted with 1 ml methanol and glass beads (0.5-mm glass beads, Scientific Industries Inc., United States) using a FastPrep cell disrupter two times for 25 s at a speed of 6.5 m s^−1^. Samples were centrifuged at room temperature for 5 min at 10 000 × g. The supernatant was collected and extraction was repeated with 1 ml of methanol.

Extracts and purified scytocyclamide methanol solutions were analyzed with UPLC-QTOF (Acquity I-Class UPLC-Synapt G2-Si HR-MS, Waters Corp., Milford, MA, USA) equipped with a Kinetex C8 column (2.1 × 50 or 100 mm, 1.7 μm, 100 Å, Phenomenex, Torrance, CA, United States). The equipment was injected with 0.5 or 1 μl samples, eluted at 40°C with 0.1% HCOOH in water (solvent A) and acetonitrile/isopropanol 1:1, + 0.1% HCOOH (solvent B) with a flow rate of 0.3 ml min^–1^. Two solvent gradients were used. 5% solvent B to 100% solvent B in 5 min, maintained for 2 min, back to 5% B in 0.50 min, and maintained for 2.50 min before next run. Alternatively, 10% solvent B to 70% of solvent B in 5 min, then to 95% of solvent B in 0.01 min, maintained for 1.99 min, then back to 10% of solvent B in 0.5 min, and finally maintained for 2.5 min before the next run. QTOF was calibrated using sodium formate and Ultramark 1621, which yielded a calibrated mass range from *m/z* 91 to 1921. Leucine Enkephalin was used at 10-s intervals as a lock mass reference compound. Mass spectral data were accumulated in positive electrospray ionization resolution mode. The MS^E^ Trap Collision Energy Ramp Started from 40.0 eV and ended at 70.0 eV.

### Bioactivity Assays

The same *S. hofmannii* PCC 7110 methanol extract used for peptide identification LC-MS was used for antimicrobial activity screening. The screening was performed with fungal and bacterial strains (Table 2). The following samples were pipetted directly on spots on agar: 50 μl cyanobacterial cellular methanol extract, 50 μl negative control (methanol), and 10 μl positive control (nystatin) (Nystatin, *Streptomyces noursei*, EMD Millipore Corp, Germany) solution 5 mg/ml in methanol for fungi and 10 μl ampicillin (Ampicillin sodium salt, Sigma, Israel) 50 mg/ml in 70% ethanol for bacteria. Solvents were allowed to evaporate, leaving the extracts diffused in the agar. Inocula were prepared by growing the fungi for 2–14 days on PDA (Potato Dextrose Agar) media at 28°C and bacteria for two days on BHI (Brain Heart Infusion) agar at 37°C. Cell mass was transferred with a cotton swab from the agar to 3 ml of sterile 5 M NaCl solution or sterile water in the case of *A. flavus*. The inocula were spread on the agar with cotton swabs. Fungal plates were incubated at 28°C and bacterial plates at 37°C for 2 days and analyzed for inhibition zones.

**TABLE 2:** Strains used in bioassays.

**Organism**	**Strain**	**Medium**	**Incubation temperature**
**Fungi**			
*Candida albicans*	FBCC 2462	PDA	28°C
*Candida guilliermondii*	FBCC 2457	PDA	28°C
*Candida krusei*	FBCC 2464	PDA	28°C
*Candida parapsilosis*	FBCC 2465	PDA	28°C
*Filobasidiella neoformans*	FBCC 2466	PDA	28°C
*Aspergillus niger*	FBCC 2467	PDA	28°C
*Aspergillus parasiticus*	FBCC 2500	PDA	28°C
*Aspergillus flavus*	FBCC 2467	PDA	28°C
**Bacteria**			
*Staphylococcus aureus*	HAMBI 66	BHI	37°C
*Enterococcus faecium*	HAMBI 1821	BHI	37°C
*Bacillus cereus*	HAMBI 1881	BHI	37°C
*Micrococcus luteus*	HAMBI 2688	BHI	37°C
*Pseudomonas aeruginosa*	HAMBI 25	BHI	37°C
*Escherichia coli*	HAMBI 1723	BHI	37°C
*Acinetobacter baumannii*	HAMBI 1760	BHI	37°C
*Enterobacter aerogenes*	HAMBI 1898	BHI	37°C
*Salmonella enterica*	HAMBI 2331	BHI	37°C

The antifungal activity of purified scytocyclamide fractions dissolved in methanol were tested with *A. flavus* performed as with the cellular extract. Disk diffusion assays were performed with purified scytocyclamides as follows. Paper disks (Blank monodiscs, Abtek biologicals Ltd., United Kingdom) were prepared with methanol solutions of the peptides, methanol as a negative control, and nystatin as a positive control. *A. flavus* inoculum was prepared as previously and spread on the plate. Disks were placed on agar and the plates were incubated at 28°C for 2 days and analyzed.

### Biosynthetic Gene Cluster Analysis

The *S. hofmannii* PCC 7110 draft genome sequence (ANNX02) was analyzed with AntiSMASH 4.1 (Blin et al., 2017) to identify the scytocyclamide biosynthetic gene clusters. AntiSMASH recognized 9 NRPS/PKS coding regions in the draft genome. The NRPS gene domain organization was compared to the scytocyclamide structure and neighboring candidate pathways for scytocyclamide biosynthesis were identified. Flanking genes with the same orientation to the NRPSs were included in the candidate biosynthetic gene cluster between 3,716,086- 3,812,822 bp. The biosynthetic gene cluster is limited from both sides by genes with opposite orientation. Adenylation domain substrate specificity prediction was performed by combining differring AntiSMASH 4.1 and AntiSMASH 5.1.2 (Blin et al., 2019) results. The scytocyclamide biosynthetic gene cluster was visualized using Artemis (Rutherford et al., 2000) and functional annotations (Supplementary Table S2) were manually refined using a combination of BLASTp and CDD database searches.

The condensation domain of NRPS module LxaC_3_ was analyzed with Natural Product Domain Seeker NaPDos (Ziemert et al., 2012) to study the role of the condensation domain in the biosynthesis of Dhb. The phylogenetic comparison was made with condensation domains with a similar position to Dhb in hassallidin biosynthesis (Vestola et al., 2014) and nodularin biosynthesis (Jokela et al., 2017) with the condensation domains of HasO_2_ and NdaA_1_, respectively.

## Results

### Structure of Scytocyclamides

**FIGURE 1:**
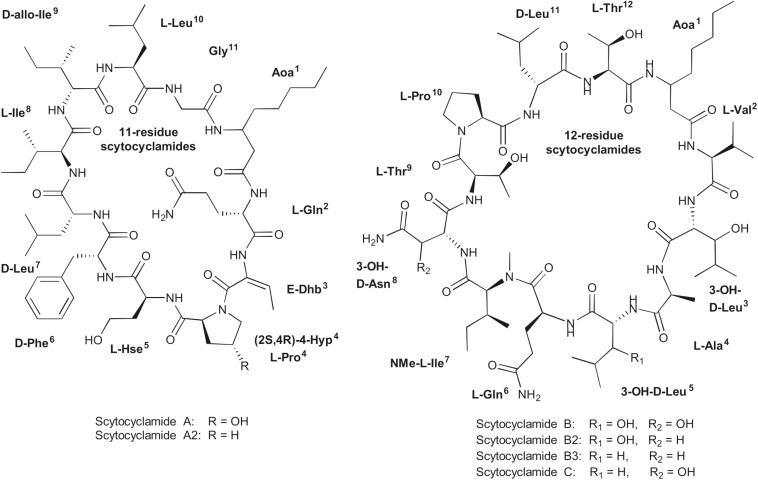
Structures of 11- and 12-residue laxaphycin variants scytocyclamides.

UPLC-QTOF analysis of *S. hofmannii* PCC 7110 methanol extract yielded six peaks corresponding to six scytocyclamide variants (Supplementary Figures S1, S2). Three of these (scytocyclamides A-C) have been previously characterized with spectrometric methods, including NMR. Three new less abundant variants, scytocyclamides A2, B2, and B3 appeared to be less hydroxylated (Figure 1, Table 3). The protonated masses and relative intensities for each compound are shown in Table 4. Product ion spectra (MS^E^) of protonated scytocyclamides A-C showed that the amino acid sequence could be generated from high-intensity ions in which Pro is N-terminal (Supplementary Figures S3, S4). Application of this fragmentation behavior to product ion spectra (MS^E^) of the new scytocyclamides A2, B2, and B3 clearly showed the amino acids lacking a hydroxyl group (Supplementary Figures S3, S4). Scytocyclamides A and A2 fall in 11-residue laxaphycins and scytocyclamides B-C fall in 12-residue laxaphycins. The yields for each compound were 1 mg (A), 1 mg (A2), 3 mg (B), 0.8 mg (C), 0.4 mg (B2), and 0.4 mg (B3).

**TABLE 3:** Structures of scytocyclamides from *S. hofmannii* PCC 7110, with new variants A2, B2, and B3.

**11-residue Laxaphycins**	**1**	**2**	**3**	**4**	**5**	**6**	**7**	**8**	**9**	**10**	**11**	
Scytocyclamide A	Aoa	L-Gln	E-Dhb	L-OHPro	L-HSe	D-Phe	D-Leu	L-Ile	D-*allo*-Ile	L-Leu	Gly	
Scytocyclamide A2	Aoa	L-Gln	E-Dhb	L-Pro	L-HSe	D-Phe	D-Leu	L-Ile	D-*allo*-Ile	L-Leu	Gly	

**12-residue Laxaphycins**	**1**	**2**	**3**	**4**	**5**	**6**	**7**	**8**	**9**	**10**	**11**	**12**

Scytocyclamide B	Aoa	L-Val	D-OHLeu	L-Ala	D-OHLeu	L-Gln	NMe-L-Ile	D-OHAsn	L-Thr	L-Pro	D-Leu	L-Thr
Scytocyclamide B2	Aoa	L-Val	D-OHLeu	L-Ala	D-OHLeu	L-Gln	NMe-L-Ile	D-Asn	L-Thr	L-Pro	D-Leu	L-Thr
Scytocyclamide B3	Aoa	L-Val	D-OHLeu	L-Ala	D-Leu	L-Gln	NMe-L-Ile	D-Asn	L-Thr	L-Pro	D-Leu	L-Thr
Scytocyclamide C	Aoa	L-Val	D-OHLeu	L-Ala	D-Leu	L-Gln	NMe-L-Ile	D-OHAsn	L-Thr	L-Pro	D-Leu	L-Thr

*Stereochemistry according to epimerase location in the biosynthetic gene cluster modules and Grewe (2005).*

**TABLE 4:** Scytocyclamides A–C from *S. hofmannii* PCC 7110.

	**t_R_**	**[M + H]^+^**			
**11-residue laxaphycins**	**(min)**	**Exp (*m/z*)**	**Δ (ppm)**	**Formula**	**RI (%)**
Scytocyclamide A	3.46	1223.7399	0,0	C_61_H_99_N_12_O_14_	98
Scytocyclamide A2	3.56	1207.7422	−2.3	C_61_H_99_N_12_O_13_	2
**12-residue laxaphycins**					
Scytocyclamide B	3.10	1367.8173	2.1	C_63_H_111_N_14_O_19_	50
Scytocyclamide B2	3.14	1351.8169	−2.0	C_63_H_111_N_14_O_18_	18
Scytocyclamide B3	3.27	1335.8228	−1.4	C_63_H_111_N_14_O_17_	10
Scytocyclamide C	3.23	1351.8190	−0.4	C_63_H_111_N_14_O_18_	22

*Retention times (t_R_), experimental (Exp) mass of protonated scytocyclamides, difference (Δ) to calculated mass, chemical formula, and relative intensity (RI) of pronated scytocyclamides.*

### Scytocyclamide Biosynthetic Gene Cluster

**FIGURE 2:**
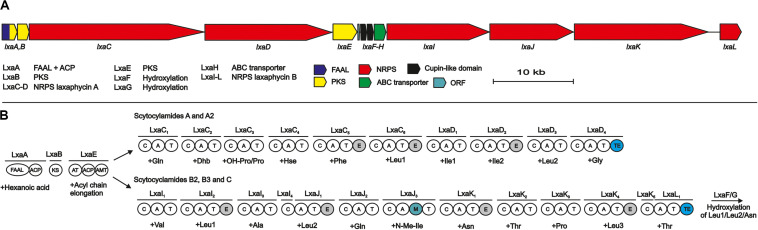
The scytocyclamide (*lxa*) biosynthetic gene cluster and putative biosynthetic scheme. **(A)** Organization of predicted scytocyclamide biosynthetic genes. **(B)** Proposed biosynthetic pathway of scytocyclamides. NRPS, Non-ribosomal peptide synthetase, PKS, Polyketide synthase, FAAL, Fatty acyl AMP Ligase, ACP, acyl carrier protein, KS, ketosynthase, AT, acyltransferase, AMT, aminotransferase, C, condensation domain, A, adenylation domain, T, thiolation domain, M, methylation domain, TE, thioesterase domain.

Analysis of the public 12.3-Mb draft genome of *S. hofmannii* PCC 7110 identified 15 putative NRPS/PKS pathways in 9 regions recognized by AntiSMASH (Table or figure reference). Two sets of NRPSs with domain architecture matching the amino acid sequences of the two scytocyclamide types were found, separated from each other by a 9-kb region encoding 5 ORFs (Figure 2). Surprisingly, just a single candidate enzyme for the initiation of the biosynthetic pathways was found encoded with the NRPS biosynthetic genes (Figure 2). Both types of scytocyclamides contain β-aminooctanoic acid (Aoa) in their structures, and we predict that the two compounds share the initiating biosynthetic enzymes for the production of Aoa (Figure 2). The 96-kb biosynthetic gene cluster encodes 13 reading frames that were annotated *lxaA-L*, and ORF1 (Figure 2, Supplementary Table S2).

The predicted biosynthesis of both scytocyclamide types is initiated by the LxaA enzyme containing FAAL and ACP domains and is predicted to activate and load a hexanoic acid (Figure 2). The hexyl group is passed to the PKS enzymes LxaB and LxaE (Figure 2). LxaB contains a single ketosynthase (KS) domain and LxaE is composed of acyl transferase (AT), ACP, and aminotransferase (AMT) domains (Figure 2). These PKS domains elongate the hexyl chain with an acyl group to octyl chain and the aminotransferase acts on the carbonyl in the β position adding the amino group (Figure 2). We predict that β-aminooctanoic acid has two alternative branched pathways, the 11- or 12-residue scytocyclamide NRPSs (Figure 2). In 11-residue scytocyclamide synthesis the LxaC-D NRPSs and in 12-residue scytocyclamides the LxaA-D NRPS enzymes incorporate the amino acids (Figure 2). Both pathways have a terminal thioesterase (TE) that head-to-tail cyclize and release the scytocyclamides. Each module of LxaC-D and LxaI-L enzymes bears a condensation (C), adenylation (A), and thiolation (T) domain (Figure 2). In addition, LxaC_5_, LxaC_6_, LxaD_2_ and LxaI_2_, LxaJ_1_, LxaJ_3_, and LxaK_4_ modules contain epimerase domains and LxaJ_3_ contains an N-methylation domain (Figure 2). The biosynthetic gene cluster encodes just a single LxaH ABC-transporter, which is characteristic of NRPS biosynthetic gene clusters.

The predicted adenylation domain substrate specificities of LxaC-D and LxaI-L match with the amino acids incorporated to scytocyclamides with some modifications (Supplementary Table S3). The scytocyclamide chemical structures contain 3-OHLeu, 3-OHAsn, 4-OHPro, and Dhb (Table 3). Scytocyclamide chemical variants with hydroxylations are the most abundant products produced by *S. hofmannii* PCC 7110 (Table 4). The Leu-binding pockets are identical (DAWFLGNVVK) for each of the four predicted Leu-activating adenylation domains (position 10 in 11-residue scytocyclamides and positions 3, 5, and 11 in 12-residue scytocyclamides) with the possible exception of a gap in the adenylation domain amino aci sequence of position 3 (—FLGNVVK) (Supplementary Table S3). Cultivation of *S. hofmannii* PCC 7110 in modified growth medium containing racemic 3-OHLeu did not result in an increase of the relative amounts of hydroxylated Leu-containing laxaphycin variants (Supplementary Figure S5). This could indicate that LxaI_2_ and LxaJ_1_ adenylation domains incorporate Leu and not 3-OHLeu, assuming that 3-OHLeu is transported into the cell. *S. hofmannii* PCC 7110 incorporated the non-proteinogenic amino acids (2S,4R)-4-MePro, (2R,4R)-4-MePro, (2S,4S)-4-MePro, (2S,4S)-4-OHPro, and (2S,4R)-4-OHPro in parallel cultivation experiments (data not shown). We predict that the cupin 8 family proteins LxaF-G hydroxylates Leu and the Asn after incorporation of the proteinogenic amino acids into the peptide intermediate by the corresponding adenylation domain (Figure 2). We did not find suitable candidate enzymes for synthesis of 4-OHPro encoded in the biosynthetic gene cluster.

The modified AA clade of condensation domains is proposed to play an active role in Thr dehydration during biosynthesis of non-ribosomal peptides (Tillett et al., 2000; Moffitt and Neilan, 2004). The Dhb-tailoring condensation domains LxaC_3_, HasO_2_, and NdaA_1_ were most similar to the modified AA clade of condensation domains in phylogenetic analysis performed using NaPDoS (Supplementary Figure S6).

### Antimicrobial Activity

**FIGURE 3:**
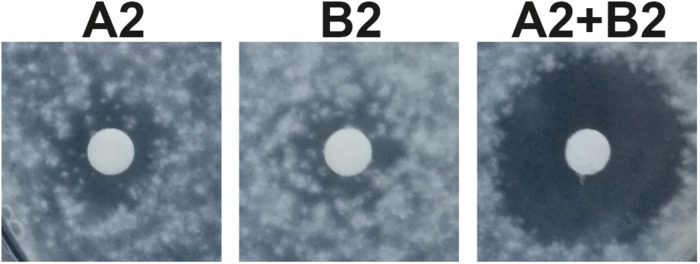
Inhibition of growth of *Aspergillus flavus* by scytocyclamides. Scytocyclamide A2 (200 μg), scytocyclamide B2 (85 μg), and scytocyclamides A2 + B2 (100 μg + 43 μg). Disk diameter is 5 mm.

Antimicrobial activity of *S. hofmannii* PCC 7110 methanol extracts was studied with several fungal and bacterial strains (Table 2). The extracts inhibited only the growth of *A. flavus* FBCC 2467. Disk diffusion assays were performed after purification of the scytocyclamides from the extract. Inhibition of fungal growth was observed with individual scytocyclamides as a hazy inhibition zone and synergy was observed between 11-residue and 12-residue compounds as a noticeably increased clear inhibition zone (Figure 3). Scytocyclamide amounts and inhibition zone diameters are shown in Supplementary Table S4. Cross-contamination between purified scytocyclamides A-D was from <1% to 5% and 15% for E (Supplementary Figure S7).

## Discussion

We described an unusual natural product biosynthetic gene cluster for producing structurally distinct scytocyclamides. Our analysis suggests that scytocyclamides have branched biosynthesis and share loading modules LxaA-B and LxaE (Figure 2 and Supplementary Table S2). These shared loading modules initiate biosynthesis to produce the β-amino acid Aoa, which is the only common amino acid in the peptide sequence of the two structural distinct types of scytocyclamides. The biosynthesis then branches to two NRPS pathways (Figure 2). The organization of the catalytic domains in the NRPS enzymes LxaC-D matches the structure of 11-residue scytocyclamides A and A2 and NRPSs LxaI-L match the structure of 12-residue scytocyclamides B, B2, B3, and C (Figure 2), as analyzed in this study and reported earlier (Grewe, 2005). Such branching is exceptional because natural product biosynthetic gene clusters are typically self-contained and act independently following the colinearity rule of PKS/NRPS biosynthesis (Guenzi et al., 1998; Callahan et al., 2009; Baral et al., 2018). However, there are other known exceptions to this rule. Modules can be skipped, as in the case of anabaenopeptin and namalide synthesis in *Nostoc* sp. CENA543, where the two compounds are produced by the same gene cluster, but a shorter product namalide is produced when three modules are skipped (Shishido et al., 2017). For example, PKS domain skipping occurs in the synthesis of leinamycin (Tang et al., 2006). Alternative starter modules have been found in the synthesis of anabaenopeptins (Rouhiainen et al., 2010) and puwainaphycins and minutissamides (Mareš et al., 2019). Gene clusters have also been shown to share enzymes in producing non-proteinogenic amino acids as in the case of anabaenopeptin and spumigin (Lima et al., 2017) and aeruginosin and spumigin, which results in the side product pseudoaeruginosin (Liu et al., 2015). Crosstalk between NRPS clusters has also been found in erythrochelin biosynthesis with two separate clusters sharing essential biosynthetic enzymes (Lazos et al., 2010). Some NRPSs incorporate multiple residues of the same amino acid iteratively, as in enterobactin synthesis (Shaw-Reid et al., 1999). Shared loading modules in laxaphycin biosynthesis are now presented as a new exception to the colinearity rule of NRPS/PKS synthesis.

Twelve-residue scytocyclamides have 3-OHLeu in positions 3 and 5 and 3-OHAsn in position 8. However, the adenylation domain substrate specificity predictions are for proteinogenic Leu and Asn with a 100% match (Supplementary Table S3). We propose that the proteinogenic amino acids act as substrates for the NRPS enzymes and the hydroxylation occurs after peptide-bond formation. We propose that hydroxylation of Leu and Asn residues in all scytocyclamides is performed by cupin 8-like proteins encoded in the biosynthetic gene cluster. The JmjC-like cupin 8 family (pfam13621) of proteins are Fe(II) or Zn(II) and α-ketoglutarate (α-KG) dependent oxygenases and act as hydroxylases and demethylases (Hewitson et al., 2002; Markolovic et al., 2016). There are examples of hydroxylation of Asn, Asp, His, Lys, Arg, and RNA in human and animal proteins (Wilkins et al., 2018). The activity of cupin 8 is specific to the amino acid position in the peptide. Their location within the biosynthetic gene cluster suggests a role in the biosynthesis of scytocyclamides. To our knowledge, this activity has not been previously reported cupin 8 proteins. The hydroxylated amino acids occur in modules with epimerase domains Figure 2. This suggests that the enzymes hydroxylating the residues are specific to D-amino acids or that the epimerase domains play a role in the hydroxylation Figure 2. Other mechanisms have previously been found to introduce 3-hydroxylated amino acids to NRPS products (Hou et al., 2011). α-KG-dependent oxygenases hydroxylate L-Arg in viomycin (Yin and Zabriskie, 2004), L-Asn in daptomycin-like peptide (Strieker et al., 2007), and D-Glu in kutzneride (Strieker et al., 2009) biosyntheses. No homologs to these enzymes were found near the scytocyclamide cluster.

Dhb is enzymatically produced from Thr recognized by the adenylation domain (Challis et al., 2000). In the case of microcystin and nodularin synthesis, the dehydration has been proposed to occur due to the active role of the following condensation domain in the process (Tillett et al., 2000; Moffitt and Neilan, 2004) and bleomycin synthesis (Du et al., 2000). These microcystin and bleomycin condensation domains have been assigned to their own clade of condensation domains as “modified AA” C-domains (Ziemert et al., 2012; Bloudoff and Schmeing, 2017). When the LxaC_3_ condensation domain was analyzed by NaPDoS, it grouped with these modified AA condensation domains (Supplementary Figure S6). The similarity of these domains with direct contact to the modified amino acid suggests that the Dhb and Dha dehydration could be indeed catalyzed by the condensation domains in these cases. For the Hse residues, no prediction was given by AntiSMASH 5.1. However, a previous version, antiSMASH 4.1.0, did recognize the corresponding binding pocket sequence for DLKNFGSDVK as Hse based on the Stachelhaus code. Hse as an amino acid in NRPS products is less common and in cyanobacteria has been previously seen in laxaphycin family peptides and nostocyclopeptide M1 (Jokela et al., 2010). However, the biosynthesis and adenylation domains for this product have not been published. OHPro has been found in other cyanobacterial natural products, such as nostoweipeptins W1-W7 and nostopeptolides L1-L4 (Liu et al., 2014). The process of incorporating the OHPro or hydroxylating the prolyl residue remain unclear.

The catalytic domain organization of the scytocyclamide gene cluster matches the laxaphycin family compound structures reported earlier. The epimerizations are conserved in 11-residue laxaphycins in positions 6, 7, and 9 and in 12-residue laxaphycins in positions 3, 5, 8, and 11. The N-methylation of the amino acid in position 7 of the 12-residue laxaphycins is also conserved. Dhb^3^ is conserved in the structures of 11-residue laxaphycins. The 3-OHLeu^3^ is conserved in 12-residue laxaphycins and 3-OHLeu^5^ and 3-OHAsn^8^ are common in 12-residue laxaphycins (Table 1). Bornancin et al. (2019) predicted that laxaphycin gene clusters should have FAAL and PKS modules to initiate biosynthesis, because the 11-residue acyclic acyclolaxaphycins have a break just before the Aoc and cyclization would be the last step of synthesis. Bornancin et al. (2015) found acyclic 11-residue laxaphycin variants with a gap between the second and third amino acid in sequence starting with the Adc. They proposed that this gap could be where the synthesis is finished and the cyclization occurs, or that the compounds they found were cleaved by environmental agents. Our results confirm the discovered acyclic 11-residue variants could be immature products of the pathway, as the linear peptide follows the biosynthetic organization we have described. With the acyclic 12-residue variants, the gap in the sequence occurs within a predicted NRPS gene and the proposed mechanism of other agents or enzymes in the environment cleaving the products would seem more reasonable.

Cyanobacteria are abundant primary producers in aquatic environments and are targeted to grazing by higher organisms, such as sea hares (Cruz-Rivera and Paul, 2007). Cyanobacteria produce a wide range of bioactive natural products (Dittmann et al., 2015; Demay et al., 2019) that seem to be produced to deter the grazing fauna in the environment (Leão et al., 2012; Mazard et al., 2016). Potential competitors to cyanobacteria are also other microbes such as chytrids, which are fungi parasitic to cyanobacteria (Agha et al., 2018). Some cyanobacterial natural products have reached clinical trials and are approved as cancer drugs (Luesch et al., 2001; Deng et al., 2013). Cyclic lipopeptides are common among the cyanobacterial natural products and typically contain a single fatty acid as in laxaphycins (Galica et al., 2017) that confers membrane-disruptive properties (Humisto et al., 2019). Laxaphycin family peptides have been shown to be toxic to or inhibit the growth of multiple organisms and cell lines (Gerwick et al., 1989; Frankmölle et al., 1992b; Bonnard et al., 1997, 2007; MacMillan et al., 2002; Maru et al., 2010; Luo et al., 2014, 2015; Dussault et al., 2016; Cai et al., 2018; Bornancin et al., 2019). We observed antifungal activity of scytocyclamides toward *A. flavus* (Figure 3 and Supplementary Table S4). In an earlier report by Grewe (2005), no activity against *C. albicans* was detected for scytocyclamides A, B, and C, which was also observed in this study. Synergistic antifungal activity between 11- and 12-residue laxaphycins has been previously reported (Frankmölle et al., 1992b; MacMillan et al., 2002). The same synergistic activity was observed between 11- and 12-residue scytocyclamides (Figure 3 and Supplementary Table S4). According to previous studies and our results, the 12-residue laxaphycins are typically more potent on their own than 11-residue laxaphycins. Our previous study on *S. hofmannii* PCC 7110 failed to identify the antifungal agent in the extract, when purified fractions lacked activity. We now conclude that the antifungal activity was most probably caused by scytocyclamides, but the purified fractions had insufficient amounts of material to produce the inhibitory effect without a synergistic partner (Shishido et al., 2015).

It is probable that the other type of laxaphycins originally existed without a synergistic partner peptide in the cells, as many laxaphycins have antimicrobial activity by themselves. Through recombination events, a synergistically acting peptide has emerged to enhance the activity of the original peptide. One possibility is that the two peptides had individual gene clusters, but the initiating enzymes have been subject to an elimination event when two distinct starter enzymes were no longer necessary. It is clear that the synergistic bioactivity and shared biosynthesis of laxaphycins go together. Similar colocalization with co-regulation of distinct synergistic biosynthetic gene clusters has been previously observed in the streptomycetal antibiotics griseoviridin and viridogrisein (Xie et al., 2012). The mechanism behind the synergistic action is usually two different compounds acting on two different targets, thus combining their activity (Caesar and Cech, 2019). It is possible that one compound makes the target cell vulnerable to the other, such as via damage to the cell wall. The colocalization of genes and shared biosynthesis suggest simultaneous regulation and expression of the synergistic products to act on a single cellular target through different mechanisms.

## Data Availability Statement

All datasets presented in this study are included in the manuscript/Supplementary Material.

## Author Contributions

LH, KS, JJ, MW, and DF designed the study. AJ, LH, and MW performed the experiments. LH, JJ, and DF analyzed and interpreted the data. LH, DF, JJ, and KS wrote the manuscript, which was corrected, revised, and approved by all authors.

## Conflict of Interest

The authors declare that the research was conducted in the absence of any commercial or financial relationships that could be construed as a potential conflict of interest.
