# Supplementary material for: Shared PKS Module in Biosynthesis of Synergistic Laxaphycins
Source: Front Microbiol. 2020 Sep 16;11:578878. doi: 10.3389/fmicb.2020.578878 (PMC7524897; doi:10.3389/fmicb.2020.578878)
Supplement: Supplementary file 1 [file Data_Sheet_1.docx]

# Supplementary material

Table S1. Recipe for modified Z8-medium lacking a source of combined nitrogen (Z8X)

| **Stock solution 1** |  |  |
| --- | --- | --- |
| MgSO_4_ · 7 H_2_O | 2.5 g |  |
| CaCl_2_ · 2 H_2_O | 3.7 g |  |
| NaCl | 32.1 g |  |
| Dissolve in 1 L of deionized water | | |
| **Stock solution 2** |  |  |
| K_2_HPO_4_ · 3 H_2_O | 2.7 g |  |
| Na_2_CO_3_ | 2.1 g |  |
| Dissolve in 1 L of deionized water | | |
| **Stock solution 3** |  |  |
| Fe-solution | FeCl_3_ · 6 H_2_O | 2.8 g in 100 ml 0.1 M HCl |
| EDTA-solution | EDTA | 3,9 g in 100 ml 0.1 M NaOH |
| Add 10 mL of Fe-solution to 900 mL of deionized water. Ad 9.5 ml of EDTA-solution and fill up to 1000 mL. | | |
| **Stock solution 4** |  |  |
| 1. | Na_2_WO_4_•2H_2_O | 0.330 g/100 mL |
| 2. | (NH_4_)_6_Mo_7_O_24_•4H_2_O | 0.880 g/100 mL |
| 3. | KBr | 1.20 g/100 mL |
| 4. | KJ | 0.83 g/100 mL |
| 5. | ZnSO_4_•7H_2_O | 2.87 g/100 mL |
| 6. | Cd(NO_3_)_2_•4H_2_O | 1.55 g/100 mL |
| 7. | Co(NO_3_)_2_•6H_2_O | 1.46 g/100 mL |
| 8. | CuSO_4_•5H_2_O | 1.25 g/100 mL |
| 9. | NiSO_4_(NH_4_)_2_SO_4_•6H_2_O | 1.98 g/100 mL |
| 10. | Cr(NO_3_)_3_•9H_2_O | 0.410 g/100 mL |
| 11. | Al_2_(SO_4_)_3_K_2_SO_4_ · 24H_2_O | 4.740 g/100 mL |
| 12. | V_2_O_5_ | 0.089 g/1000 mL |
| 13. | H_3_BO_3_ | 31.0 g /1000 mL |
|  | MnSO_4_•H_2_O | 22.3 g /1000 mL |
| Prepare trace element solution by mixing 1 mL of solutions 1-11 and 10 mL of solutions 12-13 in 700mL of deionized water. Fill up to 1000 mL with deionized water. | | |
| **Z8X** |  |  |
| Stock solution 1 | 10 mL |  |
| Stock solution 2 | 10 mL |  |
| Stock solution 3 | 10 mL |  |
| Stock solution 4 | 1 mL |  |
| Bubble 500 mL RO water with gaseous CO_2_ for 30 min. Add stock solutions 1-4 and bring to 1000 mL with reverse osmosis water. | | |

Table S2. Predicted functions of the ORFs encoded in the *lxa* cluster.

| **Protein** | **Accession number** | **Length (AA)** | **Function** | **Similarity (Protein, origin)** | **Pairwise identity** | **Accession number** |
| --- | --- | --- | --- | --- | --- | --- |
| **LxaA** | WP_017742670.1 | 715 | FAAL+ACP | PuwI *Symplocastrum muelleri* NIVA-CYA 644 | 59.72 | AXN93619.1 |
| **LxaB** | WP_017742671.1 | 474 | KS | PuwB *Anabaena minutissima* UTEX B 1613 | 71.18 | AXN93597.1 |
| **LxaC** | WA1_RS15505^a^ | 7398 | NRPS | non-ribosomal peptide synthetase *Tumebacillus avium* | 45.15 | WP_087458112.1 |
| **LxaD** | WP_017742673.1 | 5058 | NRPS | non-ribosomal peptide synthetase *Nostoc flagelliforme* | 66.91 | WP_100898072.1 |
| **LxaE** | WP_066612891.1 | 1161 | AT+ACP+AMT | PuwE [Symplocastrum muelleri NIVA-CYA 644] | 54.71 | AXN93622.1 |
| **ORF1** | WP_017742675.1 | 58 | Hypotethical protein | hypothetical protein *Scytonema* sp. UIC 10036 | 52.78 | WP_155750574.1 |
| **LxaF** | WP_017742676.1 | 296 | Cupin 8 | cupin-like domain-containing protein *Tolypothrix bouteillei* | 74.23 | WP_050045604.1 |
| **LxaG** | WP_017742677.1 | 305 | Cupin 8 | cupin-like domain-containing protein *Tolypothrix bouteillei* | 78.21 | WP_050045604.1 |
| **LxaH** | WP_017742678.1 | 595 | ABC transporter | ABC transporter ATP-binding protein/permease *Tolypothrix bouteillei* | 93.45 | WP_038095421.1 |
| **LxaI** | WP_148662958.1 | 4457 | NRPS | non-ribosomal peptide synthetase *Nostoc flagelliforme* | 64.50 | WP_100898072.1 |
| **LxaJ** | WP_148662959.1 | 3434 | NRPS | non-ribosomal peptide synthetase *Nodularia* sp. NIES-3585 | 78.88 | WP_089089654.1 |
| **LxaK** | WP_066612897.1 | 5742 | NRPS | non-ribosomal peptide synthetase *Tolypothrix bouteillei* | 80.23 | WP_050045607.1 |
| **LxaL** | WP_066612900.1 | 839 | NRPS | non-ribosomal peptide synthetase *Tolypothrix bouteillei* | 89.92 | WP_063779483.1 |
| NRPS, Non-ribosomal peptide synthetase, PKS, Polyketide synthase, FAAL, Fatty acyl AMP Ligase, ACP, acyl carrier protein, KS, ketosynthase, AT, acyltransferase, AMT, aminotransferase | | | | | | |


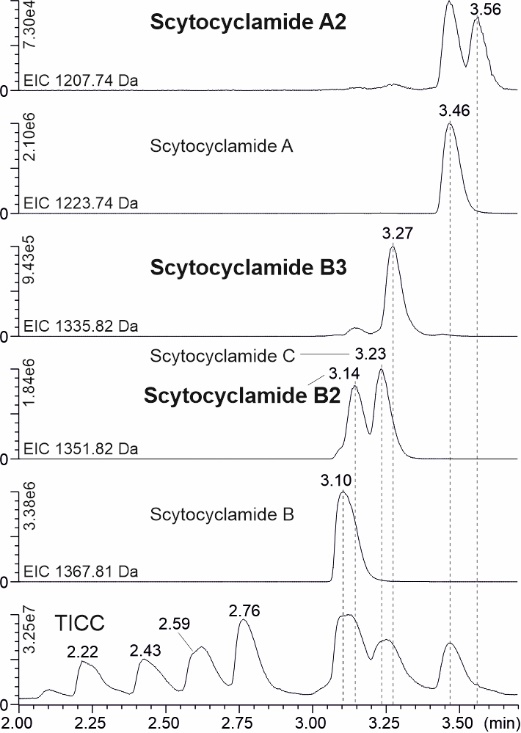


Figure S1. Extracted ion chromatograms (EIC) of scytocyclamides and total ion current chromatogram (TICC). The names of new variants are presented in bold.


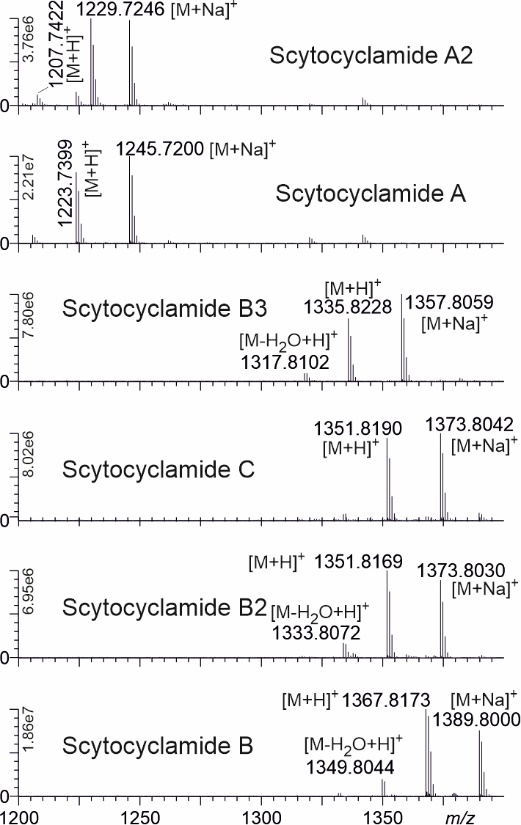


Figure S2. Mass spectra from scytocyclamide peaks with [M+H]^+^ and [M+Na]^+^ ion masses.


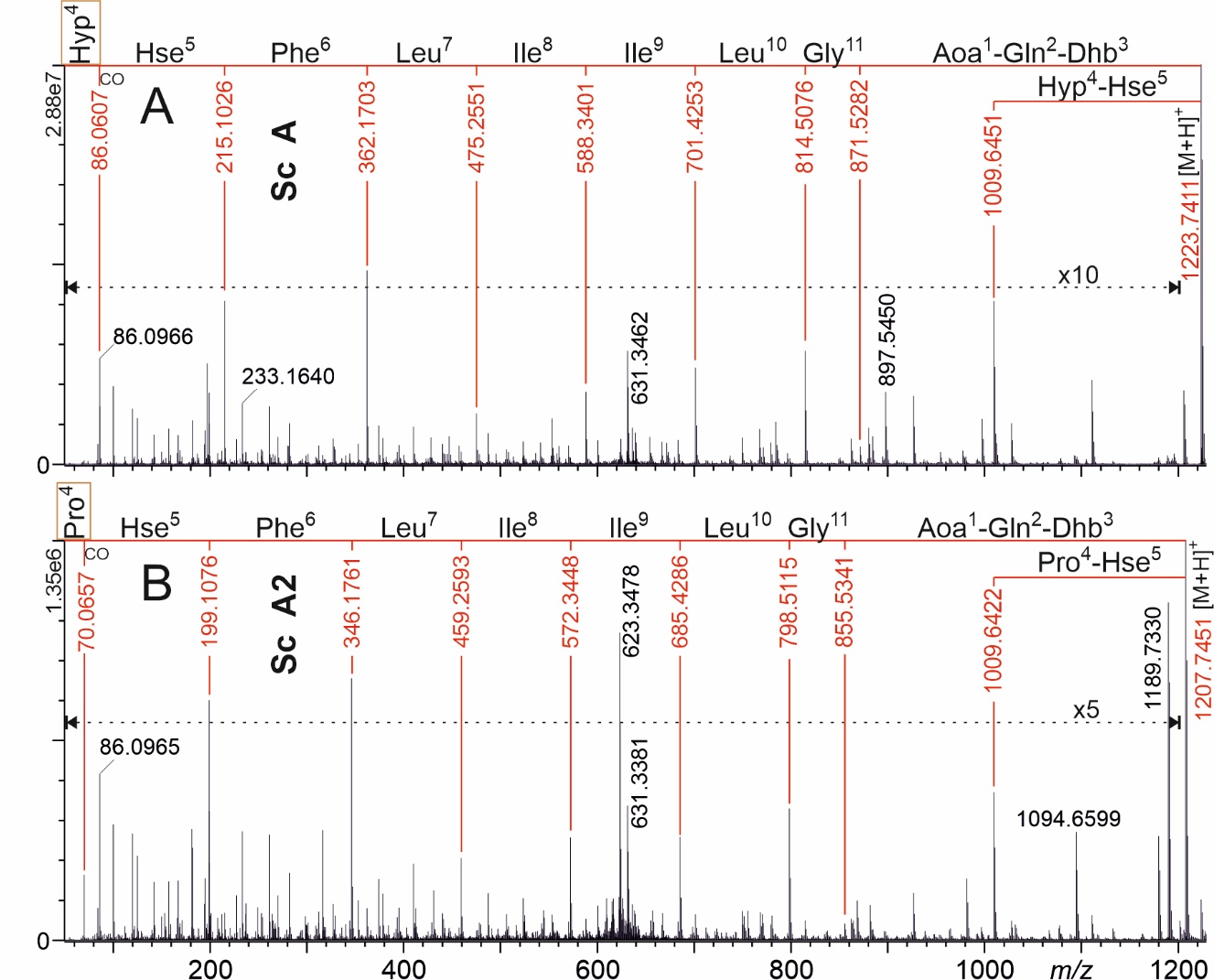


Figure S3. UPLC-QTOF product ion mass spectra of protonated 11-residue scytocyclamides (Sc) A (A) and A2 (B). The most complete product ion series showing the amino acid sequences are marked with red numbers and lines. Hyp = OHPro, Hse = Homoserine. Hydroxylated amino acids and their nonhydroxylated versions are boxed for easier recognition. Dashed horizontal lines shows the magnification (x10, x5) range of ion intensities.


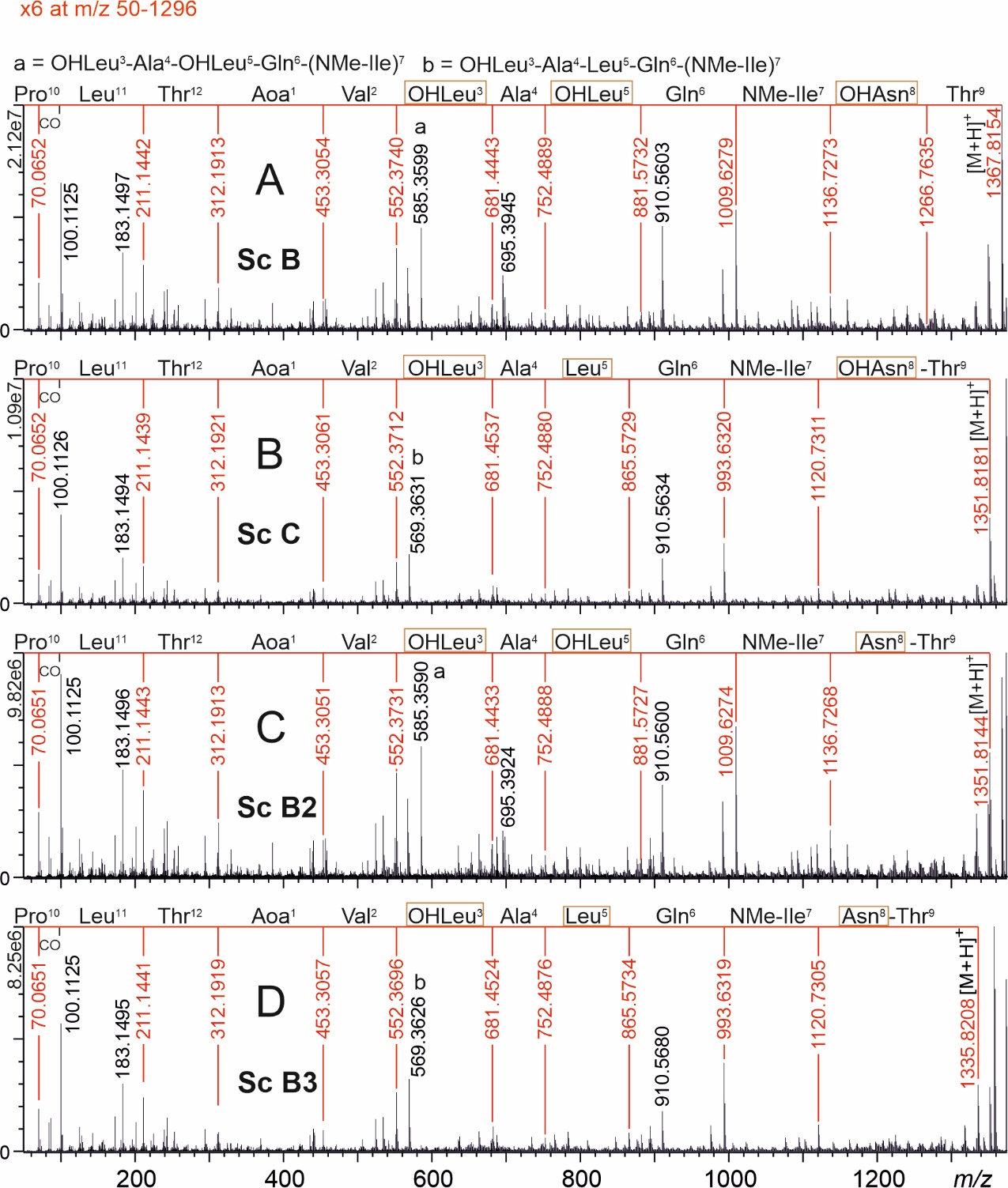


Figure S4. UPLC-QTOF product ion mass spectra of protonated 11-residue scytocyclamides (Sc) B (A), C (B), B2 (C), and B3 (D). The most complete product ion series showing the amino acid sequences are marked with red numbers and lines. Hydroxylated amino acids and their nonhydroxylated versions are boxed for easier recognition.

Table S3. Predicted and activated substrates of adenylation domains in scytocyclamides, with the binding pocket amino acid residues as identified with NRPSpredictor2 in AntiSMASH 5.1.2

| **AA residue** | **Protein** | **Predicted** | **Activated** | **A-domain residues** | **Stachelhaus code % match** |
| --- | --- | --- | --- | --- | --- |
|  | **11-residue scytocyclamides** | | | | |
| 2 | LxaC_1_ | Gln | Gln | DAWQFGLIDK | 100 |
| 3 | LxaC_2_ | Thr | Thr | DFWNIGMVHK | 100 |
| 4 | LxaC_3_ | Pro | Hyp/Pro | DAHFIAHVVK | 80 |
| 5 | LxaC_4_ | Hse* | HSe | DLKNFGSDVK | * |
| 6 | LxaC_5_ | Phe | Phe | DAWTIAAVCK | 90 |
| 7 | LxaC_6_ | Leu | Leu | DAWFLGNVVK | 100 |
| 8 | LxaD_1_ | Ile | Ile | DAFFLGVTFK | 100 |
| 9 | LxaD_2_ | Ile | Ile | DAFFLGVTFK | 100 |
| 10 | LxaD_3_ | Leu | Leu | DAWFLGNVVK | 100 |
| 11 | LxaD_4_ | Gly | Gly | DILQLGLIWK | 100 |
|  |  |  |  |  |  |
|  | **12-residue scytocyclamides** | | | | |
| 2 | LxaI_1_ | Val | Val | DAFWLGGTFK | 90 |
| 3 | LxaI_2_ | Leu | Leu | DAWFLGNVVK | 100 |
| 4 | LxaI_3_ | Ala | Ala | DLFNNALTYK | 100 |
| 5 | LxaJ_1_ | Leu | Leu | ---FLGNVVK | 70 |
| 6 | LxaJ_2_ | Gln | Gln | DAWQFGLIDK | 100 |
| 7 | LxaJ_3_ | Ile | Ile | DAFFLGVTFK | 100 |
| 8 | LxaK_1_ | Asn | Asn | DLTKIGEVGK | 100 |
| 9 | LxaK_2_ | Thr | Thr | DFWNIGMVHK | 100 |
| 10 | LxaK_3_ | Pro | Pro | DVQFMAHVVK | 90 |
| 11 | LxaK_4_ | Leu | Leu | DAWFLGNVVK | 100 |
| 12 | LxaL_1_ | Thr | Thr | DFWNIGMVHK | 100 |

*Prediction by AntiSMASH 4.1.0 based on Stachelhaus code.


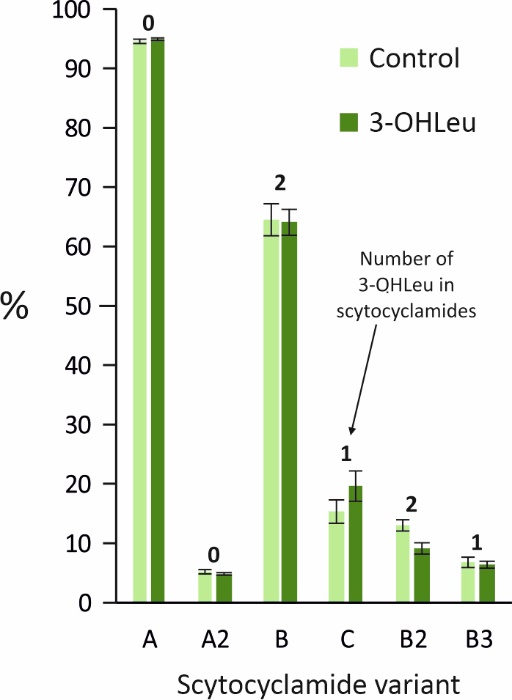


Figure S5. Relative (%) intensities of 11- (A, A2) and 12- (B, C, B2, B3) residue scytocyclamides (sum of single and doubly charged protonated and sodiated ions) from cultivations with (3-OHLeu) and without (Control) added racemic 3-hydroxyleucines in growth medium. Slight changes in the intensities can be seen, but the changes do not favor variants with more 3-OHLeu. Relative scytocyclamide intensities in Table 4 differ from these control values due to different growth conditions in the mass cultivations.


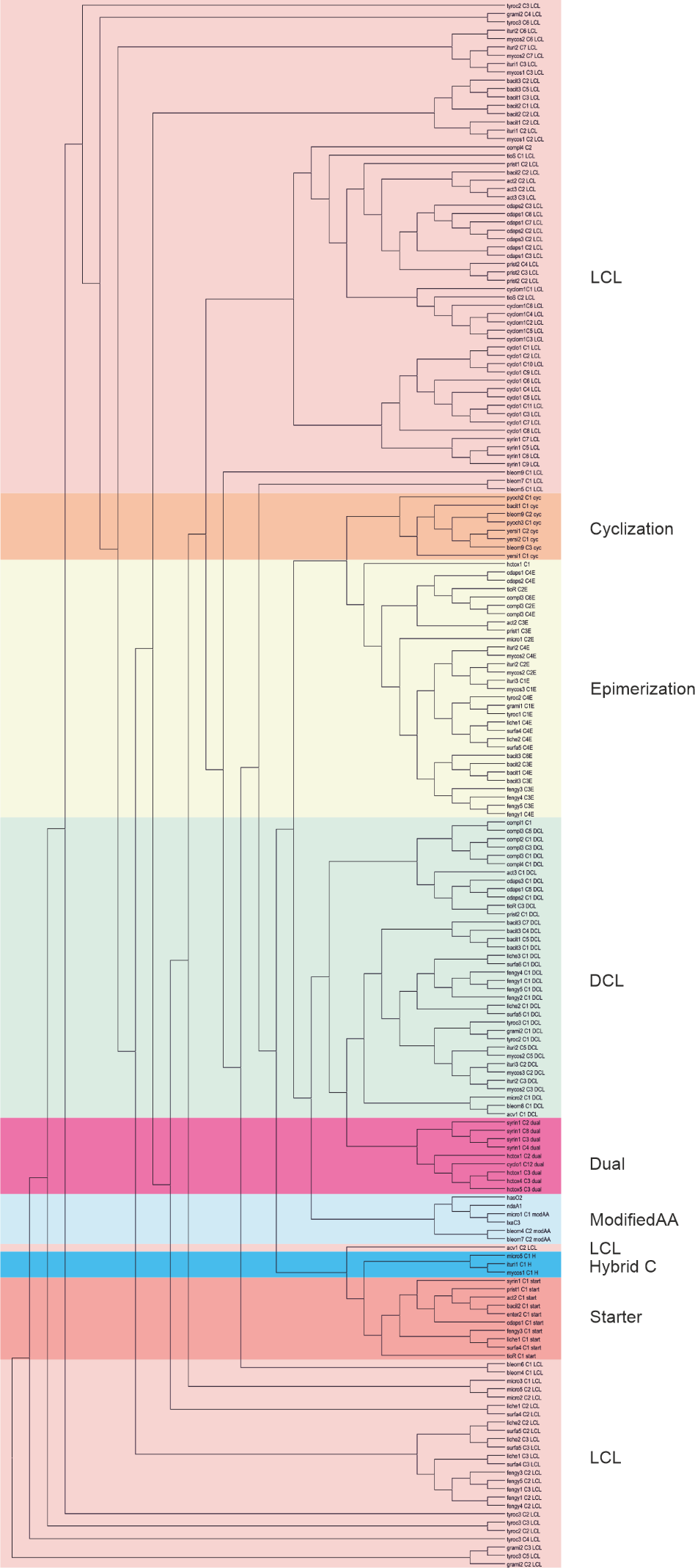


Figure S6. Phylogeny-based C-domain classification showing LxaC_3_, HasO_2_, and NdaA_1_ C-domains clustered in the modified AA clade. The phylogenetic tree was produced with NaPDoS (Ziemert et al., 2012). LCL domains catalyze a peptide bond between two L-amino acids, Cyclization domains catalyze peptide bond formation and cyclization, Epimerization domains change the chirality of the preceding amino acid, DCL domains adds an L-amino acid to the peptide ending with a D-amino acid, Dual domains catalyze both epimerization and condensation reactions, ModifiedAA domains are involved in the modification of the incorporated amino acid, Hybrid C domains are involved in the condensation of an amino acid to an aminated polyketide resulting in a hybrid PKS/NRPS product, Starter domains acylate the first amino acid with a β-hydroxy-carboxylic acid.

Table S4. Antifungal activities of scytocyclamides.

| **Compound(s)** | **Amount (µg)** | **Inhibition zone (mm)**  ***Aspergillus flavus*** |
| --- | --- | --- |
| **11-residue laxaphycins** |  |  |
| Scytocyclamide A | 200 | 10 |
| Scytocyclamide A2 | 200 | 7 |
|  |  |  |
| **12-residue laxaphycins** |  |  |
| Scytocyclamide B | 600 | 23 |
| Scytocyclamide C | 160 | 22 |
| Scytocyclamide B2 | 85 | 10 |
| Scytocyclamide B3 | 85 | 20 |
|  |  |  |
| **Synergism** |  |  |
| Scytocyclamide A+B | 100 + 300 | 36 |
| Scytocyclamide A+C | 100 + 80 | 33 |
| Scytocyclamide A2+B2 | 100 + 43 | 24 |
| Scytocyclamide A2+B3 | 100 + 43 | 25 |
| Scytocyclamide B+C | 300 + 80 | 23 |


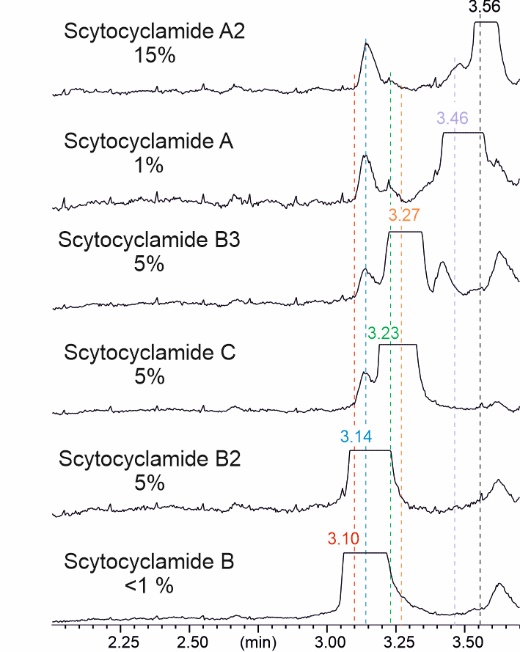


Figure S7. Total ion current chromatograms showing cross-contamination between purified scytocyclamides.
